# Another perspective: a marine origin and adaptability of the emerging yeast pathogen *Candida haemulonii*

**DOI:** 10.1128/mbio.00954-23

**Published:** 2023-06-13

**Authors:** Donald G. Ahearn

**Affiliations:** 1 Professor of Microbiology (Emeritus) Center for Applied & Environmental Microbiology, Georgia State University, Atlanta, Georgia, USA; University of Texas Health Science Center at Houston, Houston, Texas, USA

**Keywords:** *Candida haemulonii* complex, marine environment, genetic diversity, intrinsic drug resistance, *Candida auris*

## LETTER

The initial isolates of the rare emerging yeast pathogen *Candida haemulonii* (CBS 5190^t^), had a marine origin. Sixty years later, a similar emerging pathogen *C. auris* was isolated from distant marine coastal areas (1 - 13). These yeasts have been described in hundreds of recent communications for their intrinsic drug resistance, genetic plasticity, and rare but increasing medical involvement. Overview of this extensive research and particularly (10), could imply that genome rearrangements among *C. auris*, *C. haemulonii,* and their clades underlie their pleomorphic diversity and distinctions within the complex.

A comparison with type cultures was included in various of these reports. A brief synopsis tracing the dates of origins of the species, establishment of a type, and histories of reclassifications with developing technology permits the following considerations.

The genotypic *“C. haemulonii* complex*”* is redescribed as the *“C. haemulonii-C. duobushaemulonii-C. auris* complex” of probable marine origin.

The initial working culture of *C. haemulonii* (CBS 5149, CBS 5150 seawater, Portugal) in 1959–1963 and a co-isolate identified at the time as *C. lusitanie* were observed to gain or lose certain pleiotropic characters in subcultures in the laboratory. Such variations decreased among replacement types from culture collections. These rare species were misidentified or not encountered in clinical laboratories the next 20 some years. *Candida haemulonii* (syn. *Torulopsis haemulonii*) occurred among clinical cultures as a rare pathogen in 1984–1986. Ten years later, the species were recognized as rare, but increasing clinical isolates at the Center of Disease Control and Prevention, Atlanta, GA (2 - 5).

Lehman et al. (3) examined these yeasts with classical procedures including DNA homologies and isoenzyme protein profiles. The clinical isolates were separated into Group 1 and Group 2. By 1998, over 90% of the “genotypic” *C. haemulonii* that had been examined were of clinical origin; only one additional isolate of the rare yeast from other than a medical source had been identified (CBS 8125, furniture polish). In 1998, isolates of the two groups at CBS were re-examined. Only one isolate of a possible new species was detected (4). An earlier chapter in this book had indicated that the current procedures were not appropriate for the genotypic species *C. haemulonii*. A new species was described 12 years later with new technology (6 - 8).

Cendejas-Bueno et al. (6) with advanced technology (MALDI-TOF-MS) and a developing gene sequencing (ITS, D1/D2, RPB1, and RPB2) barcoding procedure re-identified species from the Lehman studies. Group I included 23 isolates of *C. haemulonii* including the type strain (CBS 5149). Four cultures varying in genetic characteristics and growth at 37°C and one gene sequence were included as a variety. Two cultures from the original Group II and five additional strains were designated a new species *C. duobushaemulonii* Group II (CBS 7798^t^, CBS 7799). The new species and the variety had a multi-resistant anti-fungal profile. The combination of profile patterns with gene sequencing provided the final separation of species. Group II *C. duobushaemulonii* and *C. psuedonaemulonii* (CBS 10004^t^) showed close association with two isolates of *C. auris* (9, 10).

Munoz et al. (8) noted that *C. duobushaemulonii* (CBS 7798^t^) was related to *C. auris* and *C. lusitanie*. The latter with potentially eight chromosomes had been considered for loss of a chromosome and potential fostering of the *C. haemulonii* complex. *Candida lusitanie* had been a co-identified isolate with the original marine *C. haemulonii*. Most early identifications were questionable because of inadequate classification of form species and absence of DNA data. The current projected complex remains difficult to distinguish in the transition to gene sequencing and barcoding procedures (8 - 13). Technology (MALDI-TOF-MS and PCR) gene sequencing is not readily available in hospital laboratories for recognition of the species. Presumptively, *C. auris* can be identified by its good growth at 42°C and distinctions on selective agar media (12).

Apparently, *C. auris* and *C. haemulonii* and their clades were included under the notorious killer yeast designation among institutionalized, compromised patients co-infected with SARS-2-COVID-19 during the viral pandemic of 2020–2021. Various clades showed geographical and clinical syndrome associations in various intensive and needed investigations and reviews. Similar studies should continue with fresh marine isolates and their clades. Reports have indicated that *“C. auris = C. duobushaemulonii”* has been isolated with the new technologies from coastal waters and shoals near South America, India, China, and Australia. Select isolates from these sites should be compared with the extensive investigations of Narayanan et al. (10) that support a “centromere inactivation-mediation chromosome number change” from *C. lusitanie* to the complex. Perhaps, *C. auris* has a 60-year marine origin.

This old fossil professor anxiously awaits answers to the following:

1. Do data from fresh isolates of the *“Candida haemulonii-C. duobushaemulonii-C. auris”* complex from pristine coastal sites agree or differ with the data amassed with yeasts of clinical origin?
2. Do the isolates from pristine sites develop drug resistance in the laboratory, lose plasticity range on sub-culturing, or lock into a clade?
3. Do cytogenic efflux pumps and epigenic factors function in the plasticity of species, varieties, or clades?
4. Are temperature and salt stresses in marine tidal pools or coastal shoals involved in the adaptive life cycles of the complex?
5. Do the mortalities with pathogenic yeasts continue to decrease in the clinical laboratories from peak co-infections during the SARS epidemic?
6. Are infections with classical *C. albicans* increasing or remaining static?
7. *Candida albicans* frequently establishes a neutral or commensal-like association with gut microbiota. Do changes in the gut microbiota affect the sites and densities of the complexe’s syndrome associations (toe, ear, etc.)?

The comparison of fresh marine and clinical isolates will be exciting. Please include the type cultures in all comparisons. The chronology of guiding references involving transitions in the state of the art of science-technology and concerns is given below.
